# From Genes to Diets: A Critical Review of Genetic, Nutrigenomic and Epigenetic Mechanisms in Type 2 Diabetes Mellitus

**DOI:** 10.3390/nu18193206

**Published:** 2026-09-28

**Authors:** Rafael G. Araújo, Gabriela Ortiz-Cruz, Sunday Sedodo Nupo, Yulianna Cordero, Lauro Cortes-Hernandez, Sandra Lucía Teniente

**Affiliations:** Center for Research in Genetics and Genomics (CIGEN), The Autonomous University of Coahuila, Saltillo 25000, Coahuila, Mexico; rafael.araujo@uadec.edu.mx (R.G.A.); ortiz.gabriela@uadec.edu.mx (G.O.-C.); sunday_nupo@uadec.edu.mx (S.S.N.); mayrecordero@uadec.edu.mx (Y.C.); laurocortes@uadec.edu.mx (L.C.-H.)

**Keywords:** type 2 diabetes mellitus, nutrigenomics, epigenetics, insulin resistance, genetic variants, artificial intelligence, precision nutrition, metabolic memory

## Abstract

Type 2 diabetes mellitus (T2D) represents over 90% of diabetes cases globally, posing a growing public health crisis with an estimated 783 million adults projected to be affected by 2045. This condition arises from a complex interplay of insulin resistance, progressive pancreatic β-cell dysfunction, and chronic low-grade inflammation, strongly influenced by genetic predisposition, obesity, sedentary lifestyle, and poor dietary habits. While genome-wide association studies (GWAS) have identified key susceptibility loci, including ***TCF7L2***, ***PPARG***, and ***KCNJ11***, these varian0ts explain only ~10% of T2D heritability, underscoring the critical role of gene–environment interactions. This review synthesizes current evidence on the genetic, nutrigenomic, and epigenetic mechanisms underlying T2D pathogenesis and progression. We examine how dietary components, such as flavonoids, terpenoids, alkaloids, and bioactive compounds from natural products, modulate gene expression via transcription factors and influence insulin sensitivity, oxidative stress, and inflammation. Furthermore, we explore the emerging role of epigenetic modifications, including DNA methylation, histone acetylation, and chromatin remodeling, in mediating the lasting effects of hyperglycemia and environmental factors on T2D risk. The potential of epigenetic biomarkers for early diagnosis, patient stratification, and personalized therapy is discussed. Finally, we highlight how next-generation sequencing and artificial intelligence, particularly machine learning and deep learning models, are advancing multi-omics data integration, enabling improved T2D subtype classification, prognosis prediction, and the development of precision nutrition and pharmacoepigenetic strategies.

## 1. Introduction

Diabetes mellitus encompasses a spectrum of metabolic disorders unified by the hallmark characteristic of persistently elevated blood glucose levels, or hyperglycemia. When hyperglycemia becomes severe, it manifests through a range of well-defined symptoms, including excessive urination (polyuria), intense thirst (polydipsia), persistent fatigue, reduced physical and cognitive performance, unexplained weight loss, blurred vision, and a heightened vulnerability to infections. If left unmanaged, these acute symptoms can escalate into life-threatening complications such as diabetic ketoacidosis or hyperosmolar hyperglycemic state, both of which carry a significant risk of coma. Beyond these acute effects, chronic hyperglycemia disrupts the normal secretion and function of insulin, leading to systemic consequences. Over time, this metabolic imbalance contributes to progressive damage and functional impairment in critical organs and tissues, including the eyes, kidneys, nerves, cardiovascular system, and blood vessels. Additionally, prolonged hyperglycemia is linked to an increased risk of developing certain types of cancer, underscoring the far-reaching and multifaceted impact of this condition on overall health [1,2,3,4].

Over the past few decades, the global prevalence of diabetes among adults has surged dramatically, rising from 4.7% in 1980 to 8.5% in 2014, with an estimated 422 million adults affected worldwide, as reported by the World Health Organization. By 2019, this figure had escalated further, with the 9th edition of the International Diabetes Federation (IDF) reporting a prevalence of 9%, affecting approximately 463 million adults globally. This escalating prevalence underscores the growing public health challenge posed by diabetes, which not only diminishes quality of life and life expectancy but also serves as a leading cause of both microvascular and macrovascular complications. Addressing the multifaceted impact of diabetes and its associated complications is therefore an urgent priority, necessitating innovative solutions and comprehensive strategies to mitigate its far-reaching consequences on healthcare systems and individual well-being [5,6].

Diabetes is classified into distinct types according to etiology and pathophysiology. Type 1 diabetes results from autoimmune-mediated destruction of pancreatic β-cells, typically leading to absolute insulin deficiency. In contrast, Type 2 diabetes (T2D) arises from a progressive decline in β-cell insulin secretion, often compounded by underlying insulin resistance. Another classification is gestational diabetes mellitus, which is diagnosed during the second or third trimester of pregnancy in women without previously evident diabetes. Additionally, there are specific forms of diabetes caused by other factors, including monogenic disorders (e.g., MODY), exocrine pancreatic diseases, and drug- or chemical-induced diabetes. In total, T2D represents the most common form of diabetes mellitus, accounting for more than 90% of all diagnosed cases. This condition imposes a substantial economic burden, with global healthcare expenditures estimated at approximately $850 billion annually. Given the significant human and financial toll of T2D, along with the difficulties in managing it effectively after diagnosis, preventive strategies have become a critical focus. For decades, researchers have proposed that modifying key risk factors could delay or even prevent the onset of T2D. The disease develops due to a combination of insulin resistance and dysfunctional insulin secretion, with contributing factors including obesity, sedentary lifestyle, and genetic susceptibility [7,8,9,10,11]. While lifestyle interventions remain pivotal in T2D prevention, emerging research underscores the need to elucidate how genetic susceptibility interacts with these modifiable risk factors to trigger disease pathogenesis. To advance our comprehension of the underlying mechanisms and refine therapeutic approaches, it is crucial to identify genetic variations that increase susceptibility to T2D. Research efforts have employed various methodologies, including candidate gene analysis, Genome-Wide Association Studies (GWAS), and advanced sequencing techniques, to uncover common, low-frequency, and rare genetic variants associated with the disease [12,13]. Beyond genetics, epigenetic mechanisms are increasingly recognized as significant contributors to T2D pathogenesis. Dysregulation of the epigenome, particularly through DNA methylation, histone modifications, and RNA-mediated gene silencing, has been linked to the onset and progression of T2D. Recent advancements have shed more light on specific genes that undergo epigenetic changes in this disease condition, highlighting the interplay between genetic and environmental influences [14,15]. Environmental factors, such as diet and lifestyle, can alter these epigenetic markers, thereby modulating gene expression and elevating the risk of T2D. Furthermore, variability in drug response among T2D patients may be attributed to differences in the expression levels of T2D-related genes, underscoring the importance of personalized treatment strategies based on genetic and epigenetic profiles [16,17].

This review aims to critically evaluate current scientific evidence on the pathophysiology of diabetes mellitus, focusing on the genetic underpinnings of T2D and the emerging roles of nutrigenomics and epigenetics in disease prevention and treatment. Specifically, it seeks to explore how the interplay between genetic, environmental, and nutritional factors modulates gene expression, offering novel insights into personalized management strategies for diabetes. Key areas of focus include: (1) the pathophysiology of T2D; (2) identification of key susceptibility genes and their regulation by environmental and dietary factors; (3) the role of nutrigenomics in modulating gene expression through dietary interventions; (4) epigenetic mechanisms and their influence on diabetes-related genes; and (5) clinical applications of nutrigenomics and epigenetics, alongside future research directions leveraging next-generation sequencing (NGS) and artificial intelligence for advanced data analysis. This review aims to provide a comprehensive foundation for developing precision-based approaches in diabetes care.

## 2. Pathophysiology of Type 2 Diabetes Mellitus

T2D is one of the most prevalent metabolic disorders worldwide. It arises from a combination of impaired insulin production by pancreatic β-cells and the failure of insulin-sensitive tissues to respond adequately to insulin. T2D is therefore characterized by insulin resistance (IR) and varying degrees of insulin secretory dysfunction, primarily due to progressive pancreatic β-cell impairment [18,19]. The molecular mechanisms governing insulin production, secretion, and action in target tissues must be tightly regulated to meet metabolic demands; consequently, dysfunction in any of these regulatory systems may lead to metabolic imbalance and the onset of T2D. This condition accounts for more than 90% of all diabetes mellitus cases and is defined by tissue insulin resistance, an inadequate compensatory insulin secretory response, and reduced insulin secretion from pancreatic islet β-cells.

A hallmark of T2D is obesity or an elevated body fat percentage, particularly central (abdominal) adiposity. In this context, adipose tissue contributes to insulin resistance through multiple inflammatory mechanisms, including increased secretion of free fatty acids (FFAs) and dysregulated adipokine production [20,21]. Obesity is strongly associated with various metabolic disorders, such as hypertension, T2D, cardiovascular disease, dyslipidemia, and certain cancers. Excess body fat is the most significant modifiable risk factor for T2D, contributing to approximately 55% of cases. In clinical settings, the prevalence of overweight and obesity among T2D patients is notably high. For instance, a study in the UK found that 86% of T2D patients were overweight or obese, with 52% classified as obese [22,23,24].

Obesity in individuals with T2D arises from a multifactorial etiology encompassing genetic, behavioral, and environmental factors. Familial aggregation of obesity and diabetes underscores the significant role of genetic predisposition in disease development. Lifestyle factors, particularly sedentary behavior and poor dietary habits, are strongly associated with weight gain and obesity. High-calorie diets rich in processed foods and sugary beverages contribute to the onset of insulin resistance and dyslipidemia, thereby accelerating the progression to T2D. Sedentary lifestyles further exacerbate this risk, with studies indicating that prolonged sedentary behavior significantly increases the likelihood of developing metabolic dysfunctions linked to obesity [25,26,27].

The prevalence of obesity among individuals with T2D is also influenced by a complex interplay of socioeconomic, psychological, and medical factors. Access to nutritious food and opportunities for regular physical activity are often limited for individuals from lower socioeconomic backgrounds, contributing to higher obesity rates in these populations [28,29]. Psychological factors such as stress, anxiety, and depression can lead to emotional eating, characterized by the consumption of high-calorie foods in response to negative emotions; this coping mechanism is associated with weight gain and increased obesity risk [30]. Certain medical conditions, including hypothyroidism and polycystic ovary syndrome (PCOS), are linked to an elevated risk of obesity due to hormonal imbalances that affect metabolism and appetite regulation [31,32].

T2D is caused by a varied mix of insulin resistance (predominantly in muscle and liver) and a reduction in the amount of insulin secreted by pancreatic β-cells. Multiple genetic, metabolic, and environmental risk factors interact to increase disease prevalence. Evidence from epidemiological studies indicates that many cases of T2D can be prevented by improving the main modifiable risk factors (obesity, low physical activity, and an unhealthy diet), even though the genetic basis for individual predisposition is strong due to non-modifiable risk factors such as ethnicity and family history/genetic predisposition [33,34]. The pathophysiology of T2D can also be linked to other alterations, emphasizing the dysregulation of hormone secretion along the entero-hypothalamic and entero-insular axis, which reflects the disease’s multifaceted nature [35].

It is now recognized that a key factor contributing to the development of diabetes is the β-cell’s inability to respond to the growing demand for peripheral insulin, a phenomenon observed throughout the progressive evolution of IR in glucose-intolerant patients. In this regard, all patients with T2D have detectable β-cell dysfunction because, although the degree of IR gradually increases over time, β-cell functional deterioration is progressive, resulting in a progressive loss of the β-cells’ ability to respond to treatment [36,37]. During the progression of IR, particularly in obese individuals, a progressive rise in basal insulin concentrations is seen. Some people can sustain this increment, while others cannot. Those who sustain it remain insulin-resistant but normoglycemic, whereas those who lose the compensatory increase inevitably lose the ability to maintain glucose homeostasis. When compared to visceral fat deposition, ectopic fat deposition has a better prognostic value and plays a more significant role in the development of IR. This occurs because fat-infiltrated muscle and liver cells interfere with their normal metabolism. Triglyceride metabolites can affect insulin signaling, glucose transport, phosphorylation, muscle glycogen synthesis, and the rise in hepatic gluconeogenesis [38].

The prevalence of diabetes as a serious health issue has skyrocketed. Nearly half a billion individuals worldwide suffer from diabetes [39]. The incidence and prevalence of T2D vary significantly by ethnicity and geographic location worldwide, with Native Americans, Japanese, and Hispanics having the highest risks. Asians have been found to have greater incidence rates than white Americans, as well as white people in the UK, where black people are at the highest risk (Figure 1) [40,41].

## 3. Genes Associated with T2D

T2D arises from a multifaceted interplay between genetic predisposition and environmental influences, many of which remain incompletely elucidated. Impaired insulin secretion and insulin resistance, culminating in chronic hyperglycemia, primarily characterize the disease [43,44]. In recent years, significant progress has been made in understanding the genetic basis of T2D. These breakthroughs have been driven by the integration of data from GWAS, the advent of high-throughput genotyping technologies, the extensive cataloging of Single Nucleotide Polymorphisms (SNPs) in publicly accessible databases, the systematic analysis of large patient cohorts, and the development of advanced computational tools for genetic analysis. However, early estimates that only ~10% of T2D heritability could be explained by a handful of loci were dramatically revised as sample sizes increased and study designs expanded to include diverse ancestral populations. Large-scale multi-ancestry meta-analyses have now mapped several hundred susceptibility loci for T2D. The DIAMANTE Consortium identified 237 loci attaining stringent genome-wide significance across 180,834 cases and 1,159,055 controls, delineated into 338 distinct association signals. Subsequent trans-ancestry meta-analyses from the Type 2 Diabetes Global Genomics Initiative, aggregating data from more than 2.5 million individuals, identified 1289 independent association signals mapping to 611 loci, 145 of which were previously unreported. More recently, Suzuki et al. (2024) [45] reported more than 600 T2D-associated loci in a meta-analysis of 428,452 cases and 2,107,149 controls. Despite this extraordinary progress, these loci collectively explain only a modest fraction of the heritable risk, with study-level heritability estimates ranging from 18% to 34% depending on ancestry and methodological approach. This persistent gap underscores the complexity of T2D’s genetic architecture and highlights the continued contribution of rare variants, gene–environment interactions, and epigenetic mechanisms not captured by common-variant GWAS. Furthermore, while multi-ancestry genetic risk scores have demonstrated superior transferability across diverse populations relative to ancestry-specific scores, ongoing efforts to refine predictive algorithms, standardize analytical pipelines, and enhance representation of African, Hispanic, and South Asian ancestry groups will further strengthen their clinical potential. A balanced appraisal of these studies also points to methodological opportunities, including addressing residual population stratification, improving imputation of low-frequency ancestry-specific variants, and advancing fine-mapping approaches to distinguish causal from linked variants, which are actively being tackled by the research community. Rather than tempering enthusiasm, these considerations serve as a constructive roadmap for future investment in diverse, deeply phenotyped cohorts, functional validation of candidate loci, and rigorous translational studies, paving the way toward the reliable integration of genetic findings into personalized diabetes prevention and management strategies [45,46].

The genetic basis of T2D is further supported by compelling evidence from population-based, familial, and twin studies. Offspring of a single affected parent face a 40% lifetime risk of developing T2D, significantly higher than the baseline population risk of 7%. This risk escalates to 70% when both parents are affected. Siblings of individuals with T2D exhibit a relative risk of approximately threefold. Notably, in monozygotic twins, the concordance rate for T2D approaches 90%, highlighting the strong genetic component of the disease. These designs provide strong and highly reproducible evidence for heritability, and the risk estimates are clinically meaningful for counseling and targeted screening. However, they also have important limitations. Family and twin studies cannot fully separate genetic effects from shared environmental and early life influences, and the ~90% monozygotic concordance may overestimate genetic contribution because monozygotic twins also share intrauterine and postnatal environments. Sibling relative risks, while robust, are nonspecific and may vary with age, sex, BMI, and ascertainment. From a clinical perspective, family history remains a cheap, accessible, and useful risk stratifier, but it should be integrated with genetic and metabolic markers rather than used in isolation. Further research is needed to unravel the remaining genetic and environmental contributors [47,48].

The genetic landscape of T2D has been progressively unraveled, with several key genes emerging as significant contributors to disease susceptibility. Among the earliest genetic variants identified was the Pro12Ala polymorphism in the ***PPARG*** gene, which encodes the nuclear receptor PPARγ. This receptor plays a critical role in adipose tissue, where it regulates the transcription of genes involved in adipogenesis and lipid metabolism. Individuals homozygous for the proline allele (Pro12) exhibit increased insulin resistance and face a 20% higher risk of developing T2D, highlighting the functional relevance of this variant in metabolic dysregulation [49,50,51,52]. Another pivotal gene in T2D pathogenesis is ***KCNJ11***, which encodes the Kir6.2 subunit of ATP-sensitive potassium channels in pancreatic β-cells. These channels are functionally linked to the sulfonylurea receptor SUR1, encoded by ***ABCC8***, and play a central role in insulin secretion. Initial meta-analyses established an association between ***KCNJ11*** variants and T2D, a finding that has been consistently replicated in subsequent studies, underscoring its importance in β-cell dysfunction [53,54].

Perhaps the most robust genetic association with T2D to date involves the transcription factor 7-like 2 (***TCF7L2***) gene. ***TCF7L2*** encodes proteins that influence insulin secretion and β-cell function, and its variants have been strongly linked to T2D across diverse populations. Four specific polymorphisms within this gene have been repeatedly associated with the disease in large-scale, multi-ethnic studies, solidifying ***TCF7L2*** as a cornerstone in the genetic architecture of T2D. Together, these findings underscore the complex interplay of genetic factors influencing insulin resistance, β-cell function, and overall metabolic homeostasis of this pathology [55,56,57,58].

GWAS has enabled the identification of additional risk genes associated with T2D, many of which play critical roles in β-cell function, insulin signaling, and the pathogenesis of obesity [45] (Table 1).

## 4. Nutrigenomics and Its Impact on Diabetes Mellitus

The use of genotypic information to guide personalized nutrition depends on a clear understanding of diet–gene interactions in health and disease. However, the effectiveness of gene regulation differs among individuals because of genetic variants, food tolerances, nutrient bioavailability, nutritional requirements, and the metabolic and biochemical interactions of nutrients and their bioactive compounds. In this context, nutrigenetics and nutrigenomics should be distinguished. Nutrigenetics analyzes the health consequences of particular allelic variants in genes involved in nutrient metabolism. Nutrigenomics, by contrast, is the nutritional application of epigenetics: it is the modern omics science that studies how nutrient molecules influence gene expression. It encompasses the concepts of “environment–DNA” and “nutrient–DNA” epigenetic interactions. In most cases, these interactions do not change the DNA sequence; instead, they act on its chemistry and modify gene expression, sometimes in a heritable manner. Several actors participate in nutrient–DNA interactions, and understanding their contributions is necessary to explain how diet and lifestyle influence human health. This framework provides a rationale for designing and prescribing individualized diets based on a person’s genetic profile, with the goal of improving prevention or treatment strategies for non-communicable diseases such as T2D, obesity, cancer, and inflammatory bowel disease [78].

Carbohydrates, proteins, and lipids are the main nutritional components because of their energy contribution, but they also provide bioactive nutrients. Lipids elicit diverse molecular responses through their regulatory effects on secondary metabolite production and gene expression. Carbohydrates trigger adaptations related to carbohydrate availability in metabolic diseases and modulate intestinal microbiota diversity. Dietary proteins influence the expression of key genes and the development of white adipose tissue. Bioactive compounds are chemical substances that can directly or indirectly induce changes in gene expression. They act at the cellular level by being metabolized through primary and secondary metabolic pathways, by altering the concentrations of intermediates and substrates, by acting as ligands for transcription factor receptors, and by exerting positive or negative effects on signaling pathways [79,80]. In the context of diabetes mellitus, these nutrient-driven epigenetic and transcriptional mechanisms may help explain interindividual variability in β-cell function, insulin resistance, and response to nutritional interventions. Dietary nutrients do not merely act as substrates for metabolism; they can also modify the availability and activity of epigenetic enzymes through nutrient-derived metabolites and cofactors, establishing a mechanistic link between nutrition, cellular metabolism, and the long-term regulation of gene expression. Dietary nutrients influence epigenetic regulation through various interconnected mechanisms, including DNA methylation, post-translational histone modifications, chromatin remodeling, and non-coding RNA expression. Among these mechanisms, one-carbon metabolism represents one of the most direct biochemical links between nutrient intake and epigenetic regulation. Nutrients such as folate, choline, betaine, and vitamin B12 contribute to the generation, recycling, or transfer of methyl groups required for cellular methylation reactions. The central metabolite connecting these pathways is S-adenosylmethionine (SAM), the universal methyl group donor for DNA, RNA, proteins, and histones [81].

Folate (vitamin B9) plays a central role in one-carbon metabolism. Dietary folate is converted into tetrahydrofolate derivatives that transport and transfer one-carbon units. Specifically, 5-methyltetrahydrofolate (5-methyl-THF) provides the methyl group required for the remethylation of homocysteine to methionine. This reaction is catalyzed by methionine synthase and requires vitamin B12 as a cofactor. Subsequently, methionine is converted into SAM via the methionine cycle. SAM donates its methyl group to various substrates through the action of DNA methyltransferases (DNMTs) and histone methyltransferases (HMTs), generating S-adenosylhomocysteine (SAH). Since SAH inhibits many methyltransferases, the balance between SAM and SAH, the cellular methylation potential, is a key determinant of epigenetic activity [82].

Choline represents another major dietary source of methyl groups. It can be oxidized to form betaine, which acts as a methyl donor via the betaine-homocysteine methyltransferase (BHMT) pathway, particularly in the liver and kidney. Betaine remethylates homocysteine to methionine independently of folate-dependent methionine synthase. Consequently, dietary choline and betaine can influence methionine availability and, in turn, alter the cellular SAM/SAH ratio. This provides a metabolic mechanism by which variations in the dietary availability of methyl donors can affect DNA and histone methylation [83]. Vitamin B12 plays a particularly important regulatory role, as it links folate metabolism with methionine metabolism. As a cofactor for methionine synthase, B12 enables the transfer of the methyl group from 5-methyl-THF to homocysteine. Therefore, B12 deficiency can impair methionine regeneration and reduce SAM availability, while simultaneously contributing to homocysteine accumulation and altered folate utilization. Thus, maintaining adequate vitamin B12 status is essential for efficient coupling between dietary folate and cellular methylation machinery. SAM-dependent DNA methyltransferases, specifically DNMT1, DNMT3A, and DNMT3B, use SAM to methylate cytosine residues, predominantly at CpG sites, resulting in the formation of 5-methylcytosine (5mC). DNA methylation can alter transcription by directly interfering with transcription factor binding or by recruiting methylated-CpG-binding proteins and chromatin-modifying complexes. In metabolic tissues including the liver, adipose tissue, skeletal muscle, and pancreas, changes in nutrient availability can modify DNA methylation in genes involved in glucose transport, lipid synthesis and oxidation, insulin signaling, inflammation, mitochondrial function, and adipocyte differentiation. Notably, these effects do not necessarily depend on the absolute intake of a single methyl donor; rather, they depend on the interplay between nutrient availability, intracellular metabolism, SAM production, SAH accumulation, cellular energy status, and the activity of methyltransferases and demethylases [81].

This same SAM pool also supplies histone methyltransferases, enzymes that methylate lysine and arginine residues on histones. Depending on the specific residue and the degree of methylation, histone methylation can promote either transcriptional activation or repression. For example, H3K4 methylation is typically associated with active chromatin, whereas H3K9 and H3K27 methylation are frequently associated with transcriptionally repressed chromatin. Consequently, alterations in one-carbon metabolism can influence not only DNA methylation but also the histone code. This is particularly relevant in metabolic tissues, as histone methylation regulates transcriptional programs controlling gluconeogenesis, fatty acid metabolism, adipogenesis, mitochondrial biogenesis, and inflammatory responses [84].

The relationship is bidirectional. While nutrients influence the epigenome, cellular metabolism also determines the availability of substrates and cofactors required by epigenetic enzymes. SAM is generated from methionine, whereas other epigenetic enzymes depend on metabolites such as acetyl-CoA, NAD^+^, α-ketoglutarate, and flavin adenine dinucleotide (FAD). These metabolites link carbohydrates, lipids, and amino acid metabolism—as well as mitochondrial metabolism—to chromatin regulation. This gives rise to an important concept: metabolic-epigenetic coupling. Nutritional status alters intracellular metabolite concentrations; these metabolites influence the activity of epigenetic enzymes, and the resulting chromatin modifications alter gene expression, which in turn remodels cellular metabolism. Thus, the epigenome functions as a nutrient-sensitive regulatory interface rather than an isolated genetic control system [85].

According to estimates from the World Health Organization (WHO), 90% of patients with diabetes have T2D, and 50% of patients with T2D have poor eating habits and inactive lifestyles. It is estimated that by 2045, the population with diabetes will increase to a staggering 783 million, which will be almost unbearable for public health systems. In recent years, significant progress has been made in diabetes treatment and clinical medicine, but these are currently based on oral hypoglycemic agents, insulin injection systems, or bariatric surgery. However, these treatments can be associated with long-term challenges that may further affect the patient’s health, such as insulin resistance and, in some cases, the potential accumulation of metabolites in vital organs like the kidneys and liver that may be linked to prolonged use of hypoglycemic drugs. Surgery, despite causing surgical risks or side effects such as stomach ulcers, venous thrombosis, and pulmonary embolism, is the treatment with the best remission rate for diabetes [86]. Metformin, sulfonylureas, and glitazones are key therapeutic agents for the treatment of T2D, as they improve metabolic control and control various circulating inflammatory mediators. Metformin suppresses glucose production in the liver, sulfonylureas increase insulin production in pancreatic β cells, and glitazones reduce the levels of CRP, PAI-1, TNF-α, and other inflammatory markers through binding to peroxisome proliferator-activated receptors (PPARs), which triggers transcriptional activity and improves insulin action by reducing the secretion of inflammatory markers [87].

Therefore, the development of new drugs, bioactive ingredients, or methods with low adverse effects, significant therapeutic effects, and novel mechanisms is in high demand. Traditional Chinese medicine (TCM) has demonstrated over centuries the use of plants or extracts for the treatment of various diseases. Currently, with technological advances and recent research, the potential of traditional medicine in the prevention and treatment of T2D has been demonstrated. There are several plants used in TCM that have shown significant effects in the treatment of diabetes through biological activity tests, such as *Rheum palmatum* L. (Polygonaceae; *Rhei Radix et Rhizoma*) and *Rheum tanguticum* (Maxim. ex Regel) Balf. (Polygonaceae; *Rhei Radix et Rhizoma*), *Coptis chinensis* Franch. (Ranunculaceae; *Coptidis Rhizoma*), *Bupleurum chinense* DC. (Apiaceae; *Bupleuri Radix*), *Rehmannia glutinosa* (Gaertn.) DC. (Orobanchaceae; *Rehmanniae Radix*), *Wolfiporia extensa* (Peck) Ginns 1984 (Polyporaceae; *Poria*), etc. [86]. Among the countless bioactive compounds that have been identified with therapeutic potential for the treatment of diabetes, they can be classified into four major groups based on their structure: flavonoids, terpenes, alkaloids, and others. Quercetin, apigenin, berberine, catalpol, oleanolic acid, crocin, and betaine are the bioactive compounds with the most significant findings and the largest number of studies. The study of natural compounds obtained from plants offers advantages in that they exhibit low toxicity and very few side effects, and they also have a high potential for treating diabetes [88]. Flavonoids are natural compounds that have been associated with numerous physiological activities, including antioxidant, hypoglycemic, anti-inflammatory, antiviral, antimicrobial, anti-cancer, and hepatoprotective effects.

They can also inhibit the generation of ROS, malondialdehyde, and free radicals. Flavonoids can lower blood glucose levels by regulating AMPK, peroxisome proliferator-activated receptor γ (PPARγ), and α-glucosidase (α-Glu) activity. Hesperidin can inhibit starch digestion and α-Glu activity, leading to decreased blood glucose levels. Flavonoids also exhibit high anti-inflammatory activity by inhibiting nuclear factor kappa-β (NF-κB) and TNF-α, or by activating adenosine 5′-monophosphate (AMP)-activated protein kinase (AMPK) [89].

Terpenoids have antioxidant, anti-inflammatory, glucose metabolism regulation, and pancreatic islet protection bioactivities. This terpene reactivity increases antioxidant enzymes in the body by scavenging free radicals and inhibiting ROS formation. They also exhibit anti-inflammatory activity by inhibiting proinflammatory cytokines, such as oleanolic acid, by reducing serum levels and gene expression of the proinflammatory cytokines IL-1β, IL-6, and TNF-α. Additionally, terpenoids such as catalpol and oleanolic acid can control glucose metabolism by inhibiting α-Glu and regulating PPARγ [90].

Alkaloids, such as betaine, can enhance oxidative stress and increase the expression of antioxidant enzymes, as well as inhibit NF-κB and regulate glucose metabolism by activating AMPK. The therapeutic effect of alkaloids against diabetes is mediated by a variety of signaling cascades and pathways, through the inhibition or stimulation of a variety of systems, such as the inhibition of the α-glucosidase enzyme, the blockade of PTP-1B, the inactivation of DPP-IV, the modulation of oxidative stress, and the increase in insulin sensitivity [91]. Dietary fibers are non-digestible carbohydrates and play an essential role in food and human nutrition. They can be classified as resistant starches, resistant oligosaccharides, lignins, and non-starch polysaccharides such as pectin, cellulose, and hemicellulose. Insoluble fiber is neither degraded nor utilized by the gastrointestinal tract, while soluble fiber is resistant to digestive enzymes but is utilized by the anaerobic gut microbiota to produce short-chain fatty acids, which exhibit diverse bioactivities such as improving insulin sensitivity or secretion, leptin release, β-cell function, mitochondrial function, and intestinal gluconeogenesis in human organs. Several studies have confirmed that short-chain fatty acids enhance the release of peptide YY (PYY) and glucagon-like peptide-1 (GLP-1) from L (enteroendocrine) cells and also promote the release of the hormone leptin in adipose tissue through the G protein receptors GPR-41 and GPR-43, which positively impacts the treatment or improvement of T2D [92,93].

Various current studies have suggested that natural products contain bioactive compounds such as vitamins, phenolic compounds, proteins, and other specific biomolecules that may contribute to the prevention and management of T2D. Curcumin, a bioactive biomolecule derived from the Curcuma longa plant, exhibits diverse bioactivities, including potential effects relevant to the prevention and management of T2D. In vitro and in vivo studies have demonstrated the antioxidant and anti-inflammatory potential of curcumin, with findings suggesting that it may delay the development of diabetes, help preserve β-cell function, reduce β-cell death, and decrease insulin resistance. It has also been reported to regulate lipid profiles and improve insulin sensitivity/secretion in diabetic animal models and, to a more limited extent, in human studies [94,95].

Procyanidins, natural phenolic compounds present in fruits, seeds, and grains, have been shown to have a high potential for preventing and controlling T2D and its complications. The effect of procyanidins is to reduce the effects caused by glucotoxicity, such as irreversible tissue and cell damage caused by insulin resistance, reduction in insulin mRNA expression, and consequent decrease in transcription or activity of transcription factors associated with insulin production. The strong antioxidant and anti-inflammatory effects of procyanidins also prevent and protect against the negative effects triggered by lipotoxicity, such as decreased viability and functionality of pancreatic β cells, and increased glucose levels and oxidative stress in pancreatic islet β-cells [96,97].

Polyphenols and NAD^+^ precursors can modulate sirtuin activity through complementary mechanisms linking dietary components to metabolic and epigenetic regulation. Resveratrol is the most extensively studied polyphenol regarding SIRT1 activation; it has been shown to promote the SIRT1-dependent deacetylation of metabolic and transcriptional regulators, including PGC-1α and FOXO proteins. Notably, resveratrol-mediated SIRT1 activation may involve both direct substrate-dependent modulation and indirect mechanisms involving AMPK and increased NAD^+^ availability [98]. Anthocyanins can also influence sirtuin signaling; cyanidin, in particular, exhibits potent SIRT6-activating activity in experimental models and increases SIRT6 protein expression in intestinal cells. Concurrently, NAD^+^ precursors—such as nicotinamide riboside and nicotinamide mononucleotide—can boost cellular NAD^+^ availability, thereby enhancing the catalytic activity of NAD^+^-dependent sirtuins. This metabolic-epigenetic axis establishes a mechanistic link between dietary bioactive compounds, cellular NAD^+^ homeostasis, histone deacetylation, transcriptional regulation, and metabolic adaptation. However, the magnitude and physiological relevance of these effects depend on tissue-specific NAD^+^ metabolism, bioavailability, concentration, and the balance between NAD^+^ biosynthesis and consumption [99,100].

Dietary fatty acids can regulate gene expression at two interconnected levels, functioning as ligands or metabolic signals for transcription factors such as PPARs and LXR/SREBP-1c. Indirectly, they modify the availability of metabolites and cofactors that control chromatin-modifying enzymes. This establishes a mechanistic link between dietary fat composition and metabolic epigenetics [101]. Saturated fatty acids (SFAs)—particularly palmitic acid—are relatively weak endogenous ligands for PPARs compared to long-chain polyunsaturated fatty acids (PUFAs). Consequently, they generally activate the PPARα-mediated fatty acid oxidation program to a lesser extent. Conversely, SFAs can promote lipogenic signaling, especially when associated with energy excess and insulin signaling. SREBP-1c is a key transcriptional regulator of de novo lipogenesis, inducing genes such as ***ACC***, ***FASN***, ***SCD1***, and ***ELOVL6***. Increased SREBP-1c activity promotes an open, transcriptionally active chromatin environment at lipogenic loci through the recruitment of transcriptional coactivators and chromatin-modifying enzymes [102]. Importantly, the effect of SFAs on chromatin is not limited to a simple, direct “SFA-to-histone-modification” relationship. Lipid overload alters cellular concentrations of metabolites such as acetyl-CoA, NAD^+^, and α-ketoglutarate, which act as substrates or cofactors for histone acetyltransferases, sirtuins/deacetylases, and demethylases. Thus, chronic exposure to SFAs can indirectly alter the epigenetic landscape through metabolic remodeling [103]. MUFAs, such as oleic acid (18:1 n-9), can interact with PPARs, although generally with less intensity than long-chain PUFAs. Therefore, they can contribute to PPAR-dependent transcription but typically elicit a weaker PPARα response than EPA, DHA, or other highly unsaturated fatty acids. MUFAs also participate in the regulation of SREBP-1c. A key difference is that PUFAs strongly suppress SREBP-1c, whereas MUFAs generally do not exert the same degree of suppression. This may favor the continued expression of genes involved in fatty acid synthesis and desaturation under certain metabolic conditions [104]. Long-chain PUFAs, particularly *n*-3 PUFAs such as EPA and DHA, can act as PPARα ligands. Ligand binding promotes the formation of the PPARα-RXR heterodimer, the recruitment of coactivators, the displacement of corepressors, and binding to PPAR response elements (PPREs). This increases the transcription of genes involved in mitochondrial and peroxisomal β-oxidation, fatty acid transport, ketogenesis, and lipid utilization. Concurrently, PUFAs repress SREBP-1c. They reduce SREBP-1c transcription and interfere with the proteolytic processing and nuclear accumulation of SREBP-1. They can also antagonize LXR activity; given that LXR normally activates the SREBP-1c promoter, this constitutes an additional mechanism for suppressing lipogenesis [105,106].

## 5. Epigenetic Role in Diabetes

Epigenetics in T2D is a rapidly evolving field that explores how non-genetic factors influence gene expression and contribute to the pathogenesis and progression of the disease. Epigenetic modifications are changes in gene expression that do not involve alterations to the underlying DNA sequence and are characterized by their potential reversibility. These modifications include DNA methylation, histone acetylation, histone methylation, phosphorylation, and chromatin remodeling, all of which play crucial roles in the regulation of gene expression in T2D. DNA methylation involves the addition of methyl groups to cytosine residues in DNA, typically leading to gene silencing. This modification has been observed in various tissues relevant to T2D, such as pancreatic islets, adipose tissue, skeletal muscle, and the liver, and is implicated in insulin resistance and β-cell dysfunction [95,96,97,98,99,100,101,102,103,104,105,106,107,108]. 

Histone acetylation and methylation are post-translational modifications that alter chromatin structure and gene accessibility. Acetylation generally promotes gene expression by loosening chromatin, whereas methylation can either activate or repress gene expression depending on the specific histone residue modified [109,110]. Phosphorylation of histones is another post-translational modification that can influence chromatin structure and gene expression, although its role in T2D is less well characterized compared to acetylation and methylation. Chromatin remodeling involves the repositioning or restructuring of nucleosomes, thereby affecting the accessibility of DNA to transcriptional machinery. This process is crucial for the regulation of genes involved in metabolic pathways and insulin signaling [111].

Recent studies have highlighted the potential of targeting epigenetic modifications for therapeutic purposes in T2D. For instance, inhibitors of bromodomains and extra terminal (BET) domain proteins, such as apabetalone, are being investigated for their ability to modulate gene expression and prevent cardiovascular complications in T2D patients [98,107,111]. Additionally, lifestyle factors such as diet and exercise have been shown to influence the epigenetic landscape, offering potential avenues for disease management [112]. Overall, the state-of-the-art understanding of epigenetics in T2D underscores the complexity of gene–environment interactions and the potential for epigenetic therapies to complement existing treatment strategies. Further research is needed to fully elucidate the mechanisms by which epigenetic modifications contribute to T2D and to develop effective interventions targeting these pathways.

Epigenetics plays a significant role in the pathogenesis and progression of diabetes mellitus, particularly T2D. In the past, the scientific literature has highlighted several key findings in this area. One related to epigenetic alterations, such as DNA methylation in pancreatic islets, has been shown to impact insulin secretion and mitochondrial function, which are critical in the development of T2D. Changes in DNA methylation at specific sites have been associated with T2D and are linked to reduced expression of genes like ***RHOT1***, which is crucial for insulin secretion and mitochondrial dynamics [113].

There is an interplay between metabolic intermediates and epigenetic modifications in T2D. Metabolic factors such as hyperglycemia can lead to epigenetic changes, including DNA methylation and histone modifications, which affect gene transcription and β-cellfunction. This interaction underscores the role of epigenetics in the disease’s pathophysiology [114].

Epigenetic signatures in metabolic tissues have been identified, such as epigenetic modifications including DNA methylation and histone changes in various tissues relevant to metabolism, such as adipose tissue, skeletal muscle, and liver. These modifications are influenced by genetic predisposition, aging, and environmental factors like diet and exercise, contributing to the development and progression of T2D [115]. It has been shown that Metformin, a common therapeutic for T2D, induces epigenetic changes that may contribute to its effects on glycemic control and inflammation. These modifications include changes in DNA methylation and histone post-translational modifications, suggesting a potential mechanism for metformin’s pleiotropic effects [116].

Another key finding is related to metabolic memory and diabetic complications. In this sense, epigenetic changes are implicated in the concept of metabolic memory, where hyperglycemia-induced epigenetic modifications persist even after glycemic control is achieved. This persistence can lead to ongoing complications, such as diabetic kidney disease, despite normalized blood glucose levels. Targeting these epigenetic changes offers potential therapeutic opportunities to mitigate long-term complications [117,118]. Also, for diabetic retinopathy, epigenetic mechanisms have been described, including miRNA dysregulation and DNA methylation linked to the disease’s progression. These modifications influence oxidative stress, inflammation, and apoptosis, which are key processes in the development of retinopathy [119].

MicroRNAs (miRNAs) are recognized as regulators of both the pathogenesis and potential biomarker/therapeutic landscape of type 2 diabetes, acting through post-transcriptional control of insulin secretion, insulin signaling, and inflammatory pathways. miRNAs are small noncoding RNAs that repress target mRNAs and form complex regulatory networks in pancreatic β-cells, adipocytes, and hepatocytes. Key islet miRNA families implicated in T2D include miR-200, miR-7, miR-184, miR-212/miR-132, and miR-130a/b/miR-152, which contribute to β-cell compensation for insulin resistance but, when dysregulated, drive β-cell impairment and reduced insulin secretion [120]. Beyond the islet, miRNAs such as miR-126, miR-375, and miR-21 modulate endothelial function and vascular complications, linking T2D to cardiovascular disease pathogenesis [121]. Broader networks also involve PI3K-Akt/insulin signaling (targeting PIK3R1, GLUT4) and NF-κB-driven inflammatory pathways (miR-146a, TNF-α, Il-6) [122].

Overall, the evidence underscores the complexity and significance of epigenetic mechanisms in diabetes mellitus, highlighting potential avenues for therapeutic intervention and the importance of understanding these processes for disease management and prevention.

### 5.1. Genetic Predisposition, Aging and Environmental Factors for the Epigenetic Changes in Diabetes

Epigenetic changes in diabetes mellitus, particularly T2D, are influenced by a combination of genetic predisposition, aging, and environmental factors, which interact in different patterns across individuals. Certain populations are more affected by these changes due to these intersecting influences. Genetic predisposition plays a significant role in populations with a high familial incidence of T2D. For instance, the Indian population has a well-documented genetic susceptibility to T2D, which is further exacerbated by rapid urbanization and lifestyle changes that alter epigenetic processes such as DNA methylation and histone modifications [123]. This genetic predisposition, coupled with environmental factors, contributes to the high prevalence of T2D in this population. Aging is another critical factor that affects epigenetic changes in diabetes. As individuals age, there is an increased likelihood of developing T2D due to age-related epigenetic modifications that impact gene expression in tissues such as skeletal muscle, adipose tissue, and pancreatic islets [15,124]. These changes can lead to impaired insulin action and secretion, contributing to the pathogenesis of T2D. Environmental factors including diet, physical inactivity, and obesity significantly influence epigenetic modifications associated with T2D. These factors can lead to alterations in DNA methylation and histone modifications, which affect metabolic pathways and insulin sensitivity [125,126]. Populations with high rates of obesity and sedentary lifestyles are particularly vulnerable to these epigenetic changes.

Diet, especially high-fat diets, has been shown to induce epigenetic changes that contribute to insulin resistance and the pathogenesis of T2D. These dietary patterns can lead to alterations in DNA methylation and histone modifications, affecting the transcriptional activity of genes involved in insulin signaling and β-cell function [126]. Nutritional overload and obesity are associated with increased DNA methylation and histone modifications, which can reduce the expression of key genes involved in insulin secretion and action, thereby exacerbating insulin resistance [127]. Specifically, high-fat diets can lead to hypermethylation of promoters of genes such as ***PPARG***, ***INSR***, ***SLC2A4***, and ***ADIPOQ***, which are crucial for insulin signaling and glucose uptake [126]. This hypermethylation correlates negatively with gene expression, contributing to insulin resistance in obese individuals [128]. High-fat diets alter histone methylation and acetylation patterns, particularly in genes like ***PPARG*** and ***SLC2A4***, which are involved in insulin action. These modifications can occur rapidly following the induction of insulin resistance and are mediated through pathways involving the SIRT family of proteins [129].

Sedentary lifestyles contribute to imbalance in energy intake and expenditure, which can lead to obesity and metabolic disorders. These lifestyle factors are associated with changes in DNA methylation patterns in skeletal muscle and adipose tissue, both relevant to glucose homeostasis [15,127]. Physical activity, conversely, has been shown to alter DNA methylation of candidate genes for T2D, potentially mitigating the risk of disease development. Obesity-induced epigenetic changes can lead to low-grade inflammation and increased reactive oxygen species (ROS) generation, further contributing to insulin resistance and metabolic dysfunction. Obesity itself is a significant modifier of the epigenome, accelerating epigenetic aging and increasing the risk of age-related diseases, including diabetes [130]. Hyperglycemia, a hallmark of diabetes, also plays a role in epigenetic alterations. It can lead to chemical modifications of DNA and histones through metabolic intermediates, affecting gene transcription and pancreatic β-cell function. Hyperglycemia-induced epigenetic changes may persist even after glucose normalization, contributing to the phenomenon of metabolic memory, which sustains diabetic complications despite glycemic control [114,118].

In summary, populations with a strong genetic predisposition, such as those in India, older individuals, and those exposed to adverse environmental factors, are most affected by epigenetic changes in T2D. These changes underscore the complex interplay between genetic, environmental, and age-related factors in the pathogenesis of T2D, as highlighted in the medical literature [114,123,125]. Overall, this genetic predisposition, in conjunction with these environmental factors and aging, significantly impacts the epigenetic landscape in diabetes, influencing disease onset and progression through modifications in DNA methylation and histone structure (Figure 2). Understanding these interactions provides insights into potential therapeutic targets and interventions for diabetes management.

### 5.2. Therapeutic Approaches Targeting Epigenetic Modifications

Recent advances in the therapeutic approaches targeting epigenetic modifications in T2D focus on modulating DNA methylation and histone modifications to influence gene expression and improve disease outcomes. DNA methylation has been implicated in the regulation of genes involved in insulin resistance and β-cell function. Therapeutic strategies aim to reverse aberrant methylation patterns to restore normal gene function [107,131].

Histone modifications, particularly acetylation and deacetylation, are crucial in regulating chromatin structure and gene accessibility. Histone deacetylase inhibitors (HDACi) have emerged as promising agents in T2D management due to their ability to enhance insulin sensitivity and β-cell function by modulating histone acetylation levels. These inhibitors target specific HDACs involved in the pathogenesis of diabetes and its complications, such as diabetic nephropathy and retinopathy, by influencing oxidative stress and inflammation pathways [132,133].

BET inhibitors, such as apabetalone, represent a novel class of epigenetic therapies being investigated for their potential in T2D treatment. BET proteins act as epigenetic readers that regulate gene transcription by interacting with acetylated histones. Apabetalone, a selective BET inhibitor, has shown promise in clinical trials for reducing cardiovascular events in patients with T2D, particularly those with low HDL cholesterol and chronic kidney disease. This drug modulates gene expression by inhibiting BET proteins, thereby impacting pathways related to inflammation and lipid metabolism [134].

Additionally, natural compounds like resveratrol have been identified as epigenetic modulators with potential therapeutic benefits in T2D. These compounds can influence DNA methylation and histone modifications, offering a complementary approach to traditional pharmacotherapy [111]. Metformin, a widely used antidiabetic drug, also exhibits epigenetic effects by modulating DNA methylation and histone modifications, contributing to its pleiotropic benefits beyond glycemic control [116].

Overall, the exploration of epigenetic therapies in T2D is expanding, with ongoing research focusing on understanding the precise mechanisms and developing targeted interventions to improve patient outcomes. These approaches highlight the potential of epigenetic modulation as a promising avenue for T2D management.

However, inhibitors such as those directed to the BET domain can have side effects that need to be carefully considered. One of the primary concerns with BET inhibitors is their impact on liver function. In a clinical trial involving apabetalone, a significant number of patients discontinued the study drug due to elevations in liver enzyme levels, indicating potential hepatotoxicity [135]. This side effect underscores the need for monitoring liver function in patients receiving BET inhibitors.

Regarding cardiovascular events, while apabetalone was hypothesized to have favorable effects on pathways related to atherothrombosis, the trial did not demonstrate a significant reduction in major adverse cardiovascular events compared to placebo [135]. This suggests that while BET inhibitors may modulate inflammation pathways, their efficacy in reducing cardiovascular events in T2D patients remains uncertain.

In terms of inflammation pathways, BET inhibitors have been shown to disrupt interactions between BET proteins and inflammatory mediators, potentially reducing the transcription of pro-inflammatory genes [136]. This mechanism could theoretically attenuate inflammation-related damage in tissues such as pancreatic β-cells, which are crucial for insulin production and glucose regulation. However, the clinical significance of these findings in the context of T2D management requires further investigation.

Overall, while BET inhibitors like apabetalone offer a novel approach to modulating epigenetic pathways in T2D, their potential side effects, particularly concerning liver function and the uncertain impact on cardiovascular events, necessitate careful consideration and further research to fully understand their therapeutic role [135,136].

miRNA-targeted therapy remains investigational but mechanistically compelling. In human islets from T2D donors, inhibition of miR-200c using an antagomir increased glucose-stimulated insulin secretion nearly threefold, since miR-200c overexpression suppresses ETV5 and disrupts insulin exocytosis [137]. Other miRNAs, such as miR-30d and miR-375, enhance insulin gene expression and β-cell proliferation/survival when experimentally restored [138,139]. Emerging delivery platforms, including exosome-based systems, are being developed to overcome the major translational barrier of achieving tissue-specific delivery of miRNA mimics or inhibitors to β-cells or adipose tissue without off-target effects [140].

The greatest barrier to translating miRNA-targeted therapies in type 2 diabetes is delivery specificity, compounded by molecular instability, off-target/immune effects, and the biological complexity of miRNA regulatory networks. Beta-cell or tissue-restricted delivery is the dominant unmet need. Even mechanistically promising strategies, such as antagomir-mediated miR-200c inhibition, cannot advance clinically because there is no validated way to confine the agent to β-cells rather than other tissues. The same limitation applies to miRNA targets aimed at improving peripheral insulin action [138]. Naked oligonucleotides are also inherently unstable, negatively charged, and hydrophilic, preventing efficient membrane diffusion and requiring a carrier system for cellular uptake [141].

Unmodified miRNA mimics and antagomirs are rapidly degraded by serum nucleases and cleared before reaching target tissue [142,143]. Synthetic oligonucleotides can also trigger innate immune activation (e.g., TLR7 activation by certain miRNA mimics), producing cytokine release and systemic toxicity that has led to early termination of at least one oncology miRNA trial (MRX34), illustrating a safety signal relevant across miRNA drug classes [144].

Individual miRNAs regulate hundreds of transcripts, and a single mRNA can be co-regulated by multiple miRNAs, producing dense, context-dependent regulatory networks. This pleiotropy makes it difficult to predict on-target versus off-target consequences of manipulating any single miRNA, and effects can even be paradoxical depending on cellular context, as seen with miR-200c showing both protective and deleterious effects depending on conditions [145].

Beyond delivery, translation is slowed by lack of standardized pharmacodynamic/biomarker endpoints, variable pharmacokinetics, and limited validation in large, multi-ethnic human cohorts, issues explicitly flagged for the miR-200 family in T2D, where antagomiR inhibition (to improve insulin sensitivity) and physiological-dose mimics (to enhance β-cell resilience) both show promise but remain unproven in humans [145]. A recent Diabetes perspective marking the 2024 Nobel Prize for miRNA discovery similarly concludes that the mechanisms controlling islet miRNA expression and strategies for their therapeutic modulation remain poorly defined, and that integrating mechanistic, single-cell, and translational approaches will be essential to advance the field [146].

### 5.3. Epigenetic Biomarkers for T2D

DNA methylation has been extensively studied, with several CpG sites identified as potential biomarkers. For instance, methylation alterations at CpG sites in genes such as ***TXNIP***, ***ABCG1***, and ***SREBF1*** have been consistently associated with T2D across different studies and populations [147,148,149,150]. These genes are involved in key metabolic pathways, including signaling and lipid metabolism, which are crucial in the pathophysiology of T2D. The study by Giri et al. identified novel CpG sites in MIR1287 and EDN2-SCMH1, which were associated with T2D risk and could be used for patient stratification based on glycemic control and dyslipidemia [147]. Additionally, methylation markers in ***ABCG1*** and ***SREBF1*** have been evaluated for their potential in stratifying patients based on insulin resistance and sensitivity, suggesting their utility in personalized risk prediction [149].

Furthermore, García-Calzón et al. explored epigenetic biomarkers associated with metformin response and intolerance, identifying specific methylation sites that could predict glycemic response and tolerance to metformin in drug-naïve patients with T2D [151]. This highlights the potential of epigenetic biomarkers in guiding treatment decisions and optimizing therapy. This is the concept of pharmacoepigenetics: the study of epigenetic biomarkers predicting therapy response. Additionally, metformin has been shown to induce epigenetic modifications, which may contribute to its therapeutic effects beyond glycemic control, including impacts on inflammation and oxidative stress [116]. This could lead to novel therapeutic approaches targeting epigenetic enzymes or pathways, offering new avenues for intervention in T2D [112].

Multiple case–control and meta-analytic studies support circulating miRNAs as diagnostic/prognostic biomarkers. A large meta-analysis and pathway-modeling study identified 16 biologically and statistically significant candidate biomarkers, prioritized for validations in this order: miR-29a-3p, miR-221-3p, miR-126-3p, miR-26a-5p, miR-503-5p, miR-100-5p, miR-101-3p, miR-103a-3p, miR-122-5p, miR-199a-3p, miR-30b-5p, miR-130a-3p, miR-143-3p, miR-145-5p, miR-19a-3p, and miR-311-3p [152]. A separate systematic review highlighted 10 consistently replicated miRNAs across independent studies (miR-126-3p, miR-223-3p, miR-21-5p, miR-15a-5p, miR-24-3p, miR-34a-5p, miR-146a-5p, miR-148a-3p, miR-30d-5p, miR-30c-5p) as leading candidates for clinical translation [148]. In a smaller case–control study, miR-122 was elevated while miR-126-3p and miR-146a were reduced in T2D and pre-diabetes, correlating with TNF-α, IL-6, and HOMA-IR, supporting a link between miRNA dysregulation, inflammation, and insulin resistance [149]. Despite promising associations, reproducibility across cohorts and standardization of pre-analytical methods remain unresolved challenges [153,154].

Despite these promising findings, challenges such as interpatient variability, cellular heterogeneity, and the need for longitudinal studies remain. The integration of epigenetic biomarkers into clinical practice requires further validation and replication across diverse populations to ensure their robustness and reliability [150,155].

### 5.4. State of the Art in Technologies for Studying Epigenetics in T2D

The current state of the art in technologies for studying epigenetics in relation to T2D involves advanced methodologies for analyzing DNA methylation and histone modifications, which are crucial for understanding the pathogenesis and progression of T2D. Recent advances have focused on improving the precision and sensitivity of these analyses, particularly in the context of identifying biomarkers and developing therapeutic approaches.

DNA methylation studies have benefited from techniques such as Methylated CpG Tandem Amplification and Sequencing (MCTA-Seq), which allows for detailed analysis of specific methylation motifs associated with T2D, such as the CGCGCGG motif. This approach, combined with fragmentation analysis, enhances the ability to work with minimal DNA quantities, such as cell-free DNA (cfDNA) in plasma, which is crucial for noninvasive biomarker discovery [156]. cfDNA approach leverages the methylation patterns of specific genes, such as ***IGFBP-1*** and ***IGFBP-2***, which have shown potential in predicting T2D risk through their hypermethylation profiles in placental tissue associated with gestational diabetes mellitus [157]. The analysis of cfDNA fragment sizes and methylation levels could provide a novel method for early detection of T2D risk, particularly in prenatal settings.

Additionally, epigenome-wide association studies (EWAS) have been instrumental in identifying genes like ***TXNIP***, which are associated with T2D and can serve as potential biomarkers for disease diagnosis and monitoring [158]. EWAS systematically studies DNA methylation variations across the genome to identify CpG sites associated with disease risk. These studies also create co-methylation networks that can stratify patients based on clinical outcomes, such as glycemic control and lipid profiles, thereby improving clinical management and treatment efficacy [147]. Also, EWAS has been used to identify methylation markers associated with diabetic complications, such as kidney disease, further highlighting their potential in risk stratification and personalized medicine [159].

MCTA-Seq, combined with fragmentation analysis, allows for precise differentiation between long and short DNA segments, enhancing the sensitivity and specificity of biomarker detection. This approach has identified several genes, including ***EP300***, ***SRC***, ***PPARG***, ***CREBBP***, and ***NCOR2***, as potential targets for T2D screening, although clinical validation is necessary to confirm their utility [156].

Another innovative approach involves whole-genome bisulfite sequencing, which offers comprehensive insights into the DNA methylation landscape of human pancreatic islets. This method has identified numerous differentially methylated regions (DMRs) associated with T2D, linking these epigenetic changes to islet dysfunction and impaired insulin secretion [160]. This technique allows for a detailed understanding of the epigenetic dysregulation in pancreatic islets, which is crucial for elucidating the pathogenesis of T2D.

Bioinformatic approaches are being utilized to address challenges such as DNA degradation during methylation studies. These methods include optimizing DNA extraction and amplification techniques, adapting bisulfite modification processes, and employing bisulfite-free methylation techniques to enhance the analysis of minimal DNA quantities [156]. Such advancements are critical for improving the precision and reliability of epigenetic studies in T2D.

Furthermore, integrating epigenomic data with metabolomics and proteomics is gaining traction as a means to deepen the understanding of disease-associated pathways. This approach can reveal associations between DNA methylation and intermediate molecular traits, offering insights into the body’s response to physiological challenges and potentially identifying novel therapeutic targets [161].

Histone modifications, particularly the role of histone deacetylases (HDACs), are being explored for their therapeutic potential. HDAC inhibitors (HDACi) are being investigated for their ability to improve insulin sensitivity, protect β-cells, and reduce complications associated with T2D [162]. These inhibitors target the regulatory processes governed by HDACs, such as oxidative stress and inflammation, which are pivotal in the pathophysiology of T2D. Therapeutic approaches targeting epigenetic modifications are also emerging, with compounds like resveratrol and vorinostat showing potential as epigenetic modulators that can influence insulin signaling and inflammation [163]. Metformin, a widely used antidiabetic drug, has been shown to exert epigenetic effects, including modifications in DNA methylation and histone proteins, which may contribute to its pleiotropic effects beyond glycemic control [116].

Overall, the integration of these advanced technologies and therapeutic strategies holds promise for enhancing the management of T2D by providing more personalized and precise approaches to diagnosis and treatment. However, further validation and clinical studies are necessary to fully establish their efficacy and applicability in routine clinical practice.

## 6. Applications of Artificial Intelligence and Machine Learning in Diagnosis

Early diagnosis of T2D is crucial for preventing and/or reducing long-term complications. The most widely used models are traditional logistic regression models; however, emerging techniques such as Machine Learning (ML) have shown improved predictive performance for handling larger and more complex datasets, identifying nonlinear patterns and hidden correlations. A pioneering study implemented the use of ML in the PIMA Indian Diabetes Dataset. This resulted from the application of Random Forest (RF), Support Vector Machine (SVM), and Naive Bayes algorithms, achieving areas under the curve (AUC) greater than 0.85, indicating robust performance in class discrimination [164].

Other studies, such as that of Karmand et al. [165], used the Fasa Adults cohort and applied multiple supervised algorithms, such as decision trees, artificial neural networks (ANNs), and SVMs. They demonstrated that ANNs offer an optimal balance between sensitivity and specificity.

The use of deep neural networks (DNNs) is a significant advance because it allows for the capture of more complex nonlinear relationships [166]. These types of architectures use additional hidden layers that improve generalization capabilities when dealing with noisy clinical data sets. Hybrid models that integrate unstructured data using Natural Language Processing (NLP) are considered in recent developments. Pina et al. [167] indicated that clinical notes and medical reports can be processed using word embeddings and transformer models to enhance the database by adding external data. This fusion improves the sensitivity of predictive models, although it also poses privacy challenges and requires advanced data identity protection techniques.

In summary, T2D diagnosis using machine learning techniques has evolved rapidly, moving from the use of traditional supervised algorithms to sophisticated models such as deep neural networks and NLP, bringing with it a consistent improvement in predictive performance metrics.

### 6.1. Identification of T2D Subtypes and Prognosis Prediction

T2D is clinically known as a heterogeneous disease; there are clinical subtypes with different progression profiles and responses to treatment. Ahlqvist et al. [168] were pioneers in the use of K-means clustering on variables, and they identified five distinct T2D subtypes in Swedish and Finnish populations. Their analysis grouped patients into different categories, including severe autoimmune diabetes, severe insulinopenic diabetes, severe insulin-resistant diabetes, moderate obesity, and advanced age.

Each subtype showed clinically significant differences in terms of risk of complications; for example, the insulin-resistant subtype was found to have a higher cardiovascular risk, while the severely insulinopenic subtype presented increased risk of developing retinopathy.

Mizani et al. [169] recently extended this approach using Random Forest and Mixed Models to improve the robustness of predictions from electronic health records.

The use of survival analysis, mainly Cox proportional hazards models enhanced with machine learning, such as CoxBoost, has improved the prediction of long-term complications in specific patient subgroups.

These types of studies highlight the importance of interpretable ML models for classifying clinically relevant subtypes, such as cardiovascular risks, chronic kidney disease, and diabetic neuropathies. They also provide more precise tools that can be used to personalize therapeutic interventions.

### 6.2. Epigenetics and Sequencing Technologies in T2D Identification

The incorporation of multi-omics datasets has enabled more in-depth analyses of genetic regulation associated with T2D. Fraszczyk et al. [170] conducted epigenomic association studies (EWAS) on five European cohorts and were able to identify methylation patterns at hypermethylated CpG sites in the TXNIP and ABCG1 genes, which are significantly associated with the risk of developing T2D.

In the field of transcriptomics, Lv et al. [171] performed RNA-Seq studies in peripheral blood from patients with T2D who presented symptoms of excessive thirst and fatigue. They identified 112 differentially expressed genes, with enrichment in pathways related to glucose metabolism and insulin resistance. Another notable strategy has been the analysis of small non-coding RNAs (miRNAs) due to their potential as a promising source of predictive biomarkers. Alamro et al. [172] used ML-based feature selection methods to identify a panel of 12 miRNAs critical for T2D-Alzheimer’s comorbidity. Multi-omics integration goes beyond epigenomics and transcriptomics; proteomics and metabolomics have emerged as crucial tools. More recent studies combine metabolomic profiles with genomic data to improve T2D prediction. The fusion of this type of multi-omic data provides a comprehensive view of the disease and accelerates the identification of more robust predictive biomarkers.

### 6.3. Integrating AI and Omics Data into Personalized Strategies

The integration of multi-omics data using AI techniques has revolutionized the development of personalized therapies for T2D. Currently, the traditional approach to T2D treatment is being replaced by personalized therapy strategies. Wu et al. [173] combined protein-protein network analysis and machine learning to identify the therapeutic effect of celastrol. The model used, based on Support Vector Machines (SVM-RFE), selected the 50 most relevant genes and then built a classifier with an AUC of 0.89, identifying key metabolic pathways associated with the action of celastrol. Other approaches integrate genomic GWAS and metabolomic data using deep learning algorithms, thereby improving the predictive capacity of drug response. The architectures used for this are autoencoders to reduce dimensionality and optimize the most relevant features [174]. As summarized in Figure 3 the proposed AI- and ML-driven workflow for type 2 diabetes follows four sequential phases. It begins with the integration of multimodal clinical and molecular data, which are then processed through a computational toolbox of supervised and unsupervised learning, natural language processing, and survival models. This analysis feeds into two converging pathways: one generating clinically actionable outcomes such as early diagnosis, subtyping, and biomarker discovery, and the other predicting drug response. Both pathways ultimately converge on precision medicine, enabling improved patient stratification and targeted therapeutic strategies (Figure 3).

### 6.4. Artificial Intelligence and Machine Learning Tools in Genomics

The rapid advancements in next-generation sequencing (NGS) technologies have driven the need for robust computational tools that can analyze, interpret, and extract knowledge. Artificial intelligence (AI) and machine learning (ML) are of great help due to the solutions they offer, as illustrated by the tools listed in Table 2. They provide methods that can be used for the prediction of genetic variants, gene expression analysis, association of variants with phenotypes, and the development of personalized diagnostic and prognostic models.

## 7. Ethical and Technical Challenges

Despite recent advances, the application of AI and omics technologies in T2D presents numerous ethical and technical challenges. Bias in training data can result in models that perpetuate existing inequalities. Genomic data privacy remains a major concern. Even with anonymization techniques, there is a risk of re-identification, especially when combining multiple omics datasets. From a technical perspective, the lack of standardization in methods for generating, preprocessing, and analyzing multi-omics data limits their reproducibility. Furthermore, deep learning models require a large amount of labeled data, a resource that can be scarce in clinical studies. More robust regulatory frameworks are needed to strike a balance between the rapid technological advancement with the protection of patient rights.

The future of T2D research is being directed toward the development of large-scale, multi-omics data platforms integrated with AI. Personalized patient-based computational models promise to revolutionize predictive medicine. Blockchain integration could resolve privacy and security issues related to genomic data, while advances in third-generation sequencing technologies will improve the quality and quantity of data available for predictive modeling. The convergence of AI, ML, and omics technologies represents a unique opportunity to transform the clinical management of T2D. Data-driven, personalized strategies can not only improve diagnosis and prognosis but also optimize therapeutic interventions, paving the way for true precision medicine.

## 8. Conclusions and Future Perspectives

### 8.1. Conclusions

The global burden of Type 2 Diabetes Mellitus continues to escalate at an alarming rate, demanding a paradigm shift in how we understand, prevent, and manage this multifaceted metabolic disorder. This review critically appraises the current evidence on the interplay between genetic predisposition, epigenetic modifications, and environmental factors, particularly dietary components, in the pathogenesis and progression of T2D. The principal strength of the genetic evidence lies in the reproducibility of large-scale GWAS and meta-analyses, which have consistently identified hundreds of susceptibility loci and provided robust mechanistic anchors such as ***TCF7L2***, ***PPARG***, and ***KCNJ11***. However, these loci collectively explain only a modest fraction of heritable risk, with most common variants exerting small effects. Their clinical relevance for individual risk prediction therefore remains limited, and their translation into routine care is constrained by incomplete ancestry representation, residual population stratification, and unresolved functional mechanisms. The genetic architecture of T2D, while substantial, accounts for only part of heritable risk, underscoring the critical importance of gene–environment interactions.

Epigenetic mechanisms, including DNA methylation, histone modifications, and chromatin remodeling, offer a biologically plausible bridge between environmental exposures and disease phenotype. Their strengths include dynamic responsiveness, tissue specificity, and potential reversibility, which make them attractive both as mechanistic mediators and as candidate biomarkers. Nevertheless, the current evidence is limited by frequent cross-sectional designs, small sample sizes, heterogeneous tissues and assays, and insufficient standardization. The concept of metabolic memory, in which hyperglycemia-induced epigenetic alterations persist despite glycemic normalization, is compelling and clinically important because it supports early intervention. Yet causality, reproducibility across populations, and clinical actionability remain incompletely established. Epigenetic markers should therefore be regarded as promising but not yet validated for routine risk stratification or treatment guidance.

Nutrigenomics offers a transformative framework for personalized dietary interventions. Bioactive compounds from natural sources, including flavonoids, terpenoids, and alkaloids, demonstrate significant potential in modulating insulin sensitivity, reducing oxidative stress, and attenuating chronic inflammation through their effects on transcription factors and signaling pathways. The ability of specific dietary components to influence epigenetic marks suggests that nutrition can serve as a powerful tool for reshaping the epigenome and mitigating disease risk. However, the clinical relevance of these findings is still limited by small and short-term human trials, heterogeneity in populations and interventions, poor bioavailability of many compounds, and confounding by overall diet and lifestyle. Rigorous validation through well-designed randomized controlled trials, standardized outcome measures, and reliable biomarkers for patient stratification is required before personalized nutritional recommendations can be implemented.

The emergence of high-throughput sequencing technologies and artificial intelligence has revolutionized our capacity to integrate multi-omics data, identify novel biomarkers, and develop predictive models for T2D diagnosis and prognosis. Machine learning algorithms have demonstrated encouraging performance in identifying disease subtypes, predicting complications, and informing treatment selection. The convergence of genomics, epigenomics, transcriptomics, proteomics, and metabolomics within AI-driven frameworks promises to deliver clinically actionable insights. However, many models remain retrospective, single-center, and insufficiently externally validated; risks of overfitting, data shift, and limited interpretability are common. Reproducibility is further constrained by inconsistent data sharing, variable preprocessing pipelines, and lack of prospective clinical validation. AI should therefore be viewed as a powerful investigational tool rather than a ready replacement for clinical judgment.

The trajectory of T2D research and clinical management is poised for transformative advances that will fundamentally reshape our approach to this global health challenge. At the heart of these developments lies the interdisciplinary convergence of systems biology, artificial intelligence, and precision medicine, which together offer unprecedented opportunities to unravel disease complexity and deliver individualized care. Realizing this potential will depend less on technological novelty alone and more on rigorous validation, transparent reporting, and demonstrable clinical benefit.

### 8.2. Future Perspectives

The future of T2D research will be defined by the seamless integration of multi-omics data, encompassing genomics, epigenomics, transcriptomics, proteomics, and metabolomics, through sophisticated computational platforms. Large-scale, longitudinal cohort studies incorporating comprehensive multi-omics profiling will be essential for elucidating the dynamic interactions between genetic predisposition, epigenetic modifications, and environmental exposures over the life course. The development of reference epigenomes for metabolically relevant tissues, coupled with single-cell epigenomic technologies, will provide unprecedented resolution of disease mechanisms and enable the identification of regulatory networks specific to cell type. These advances will be propelled by advanced deep learning architectures, including attention mechanisms and graph neural networks, which will enhance our capacity to model complex biological networks and predict disease trajectories. The integration of electronic health records with multi-omics data through natural language processing and federated learning approaches will enable robust, generalizable predictive models while addressing critical privacy concerns. Explainable AI frameworks will be crucial for translating model predictions into clinically interpretable insights, bridging the gap between computational discoveries and actionable clinical decisions. Crucially, future studies must prioritize external validation, prospective testing, code and data sharing, and prespecified analytical protocols to ensure reproducibility and clinical relevance.

Concurrently, the development of validated nutrigenomic biomarkers will enable the design of personalized dietary interventions tailored to individual genetic and epigenetic profiles, moving beyond the current one-size-fits-all nutritional recommendations. Randomized controlled trials evaluating the efficacy of genotype-guided nutritional interventions are urgently needed to establish evidence-based guidelines that can be integrated into clinical practice. This personalized approach extends to pharmacoepigenetics, where the identification of epigenetic biomarkers predictive of drug response will facilitate the optimization of therapeutic strategies, minimize adverse effects while maximizing efficacy. The recognition that epigenetic modifications are reversible presents particularly exciting opportunities for therapeutic intervention. The development of specific, tissue-targeted epigenetic modulators, including HDAC inhibitors and BET inhibitors, could enable the restoration of normal gene expression patterns in metabolically relevant tissues, though careful evaluation of safety profiles remains essential. Combination approaches integrating epigenetic therapies with conventional pharmacotherapy and lifestyle interventions may offer synergistic benefits, while early epigenetic intervention to prevent metabolic memory could dramatically reduce the long-term complications of T2D. At present, however, these strategies remain in preclinical or early phase, and their clinical relevance should not be overstated until safety, specificity, and durable efficacy are demonstrated in adequately powered trials.

The translation of these advances into clinical practice must be accompanied by parallel progress in biomarker development and early detection strategies. The identification of robust epigenetic biomarkers in accessible tissues, including cell-free DNA methylation signatures in blood, holds promise for noninvasive early detection and risk stratification. Longitudinal studies characterizing the temporal dynamics of epigenetic changes preceding T2D onset will enable the development of predictive algorithms for primary prevention, potentially shifting the focus from disease treatment to disease prevention. The incorporation of these biomarkers into routine clinical practice, supported by cost-effective technologies, could transform population-based screening and prevention strategies, particularly in high-risk populations. However, realizing this vision requires careful consideration of ethical, legal, and social implications. Ensuring equitable access to precision medicine approaches, particularly for underserved populations disproportionately affected by T2D, is imperative. Robust regulatory frameworks, data privacy protections, and strategies for managing the psychological impact of genetic risk information must be developed in parallel with technological advances. Educational initiatives for healthcare providers and patients will be essential for the successful implementation of precision diabetes care, fostering a healthcare environment where genomic and epigenetic knowledge is integrated into routine clinical decision-making.

In conclusion, the convergence of genetic, epigenetic, nutrigenomic, and computational approaches represents a paradigm shift in our understanding and management of Type 2 Diabetes. The path forward demands interdisciplinary collaboration, rigorous validation, and a commitment to translating scientific discoveries into meaningful improvements in patient outcomes. By embracing the complexity of T2D and leveraging emerging technologies in a coordinated, ethically responsible manner, we stand poised to realize the vision of precision medicine, delivering the right intervention to the right patient at the right time, ultimately reducing the global burden of this devastating disease.

## Figures and Tables

**Figure 1:**
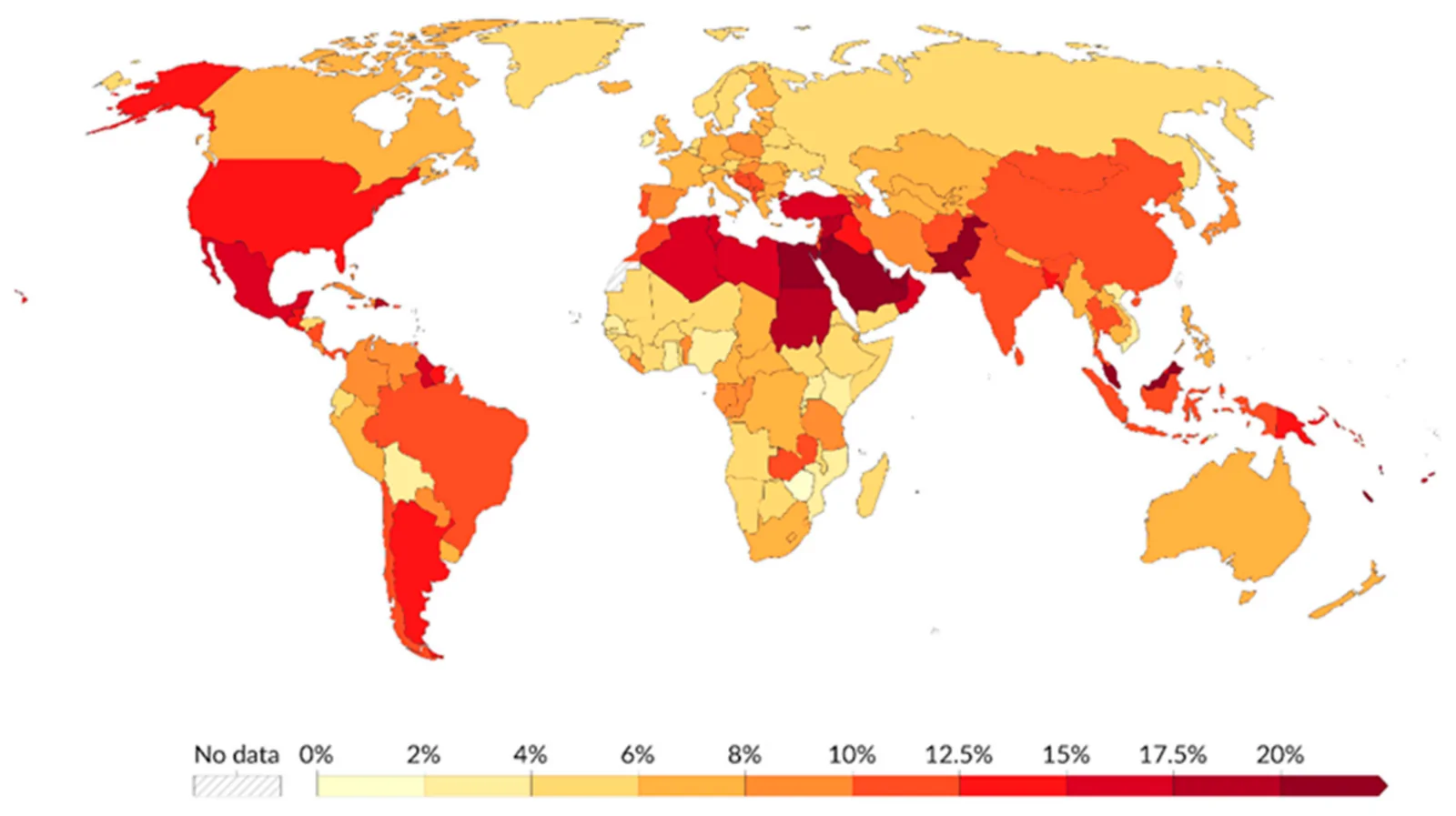
Share of people aged 20–79 with diabetes, 2024. International Diabetes Federation, via World Bank [42].

**Figure 2:**
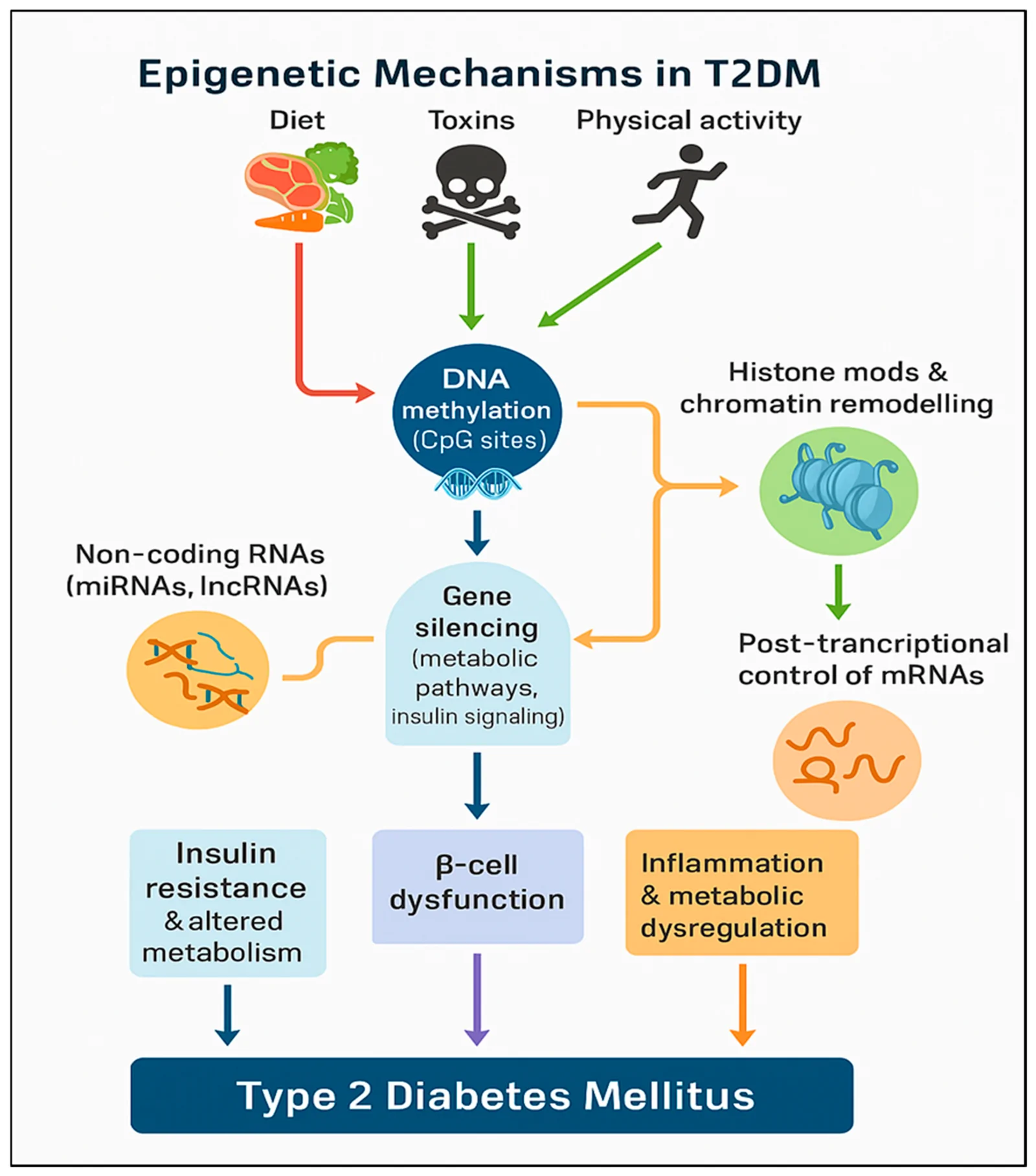
Epigenetic Mechanisms Linking Environmental Inputs to T2D. This diagram illustrates the interplay between environmental factors—diet, toxins, and physical activity—and epigenetic modifications that contribute to the pathogenesis of T2D. Environmental inputs influence three major epigenetic mechanisms: DNA methylation (blue), histone modifications and chromatin remodeling (green), and non-coding RNAs including miRNAs and lncRNAs (orange). These modifications regulate gene expression through gene silencing, transcriptional dysregulation, and post-transcriptional control, respectively. The downstream effects include insulin resistance, β-cell dysfunction, and chronic inflammation, culminating in the development and progression of T2D.

**Figure 3:**
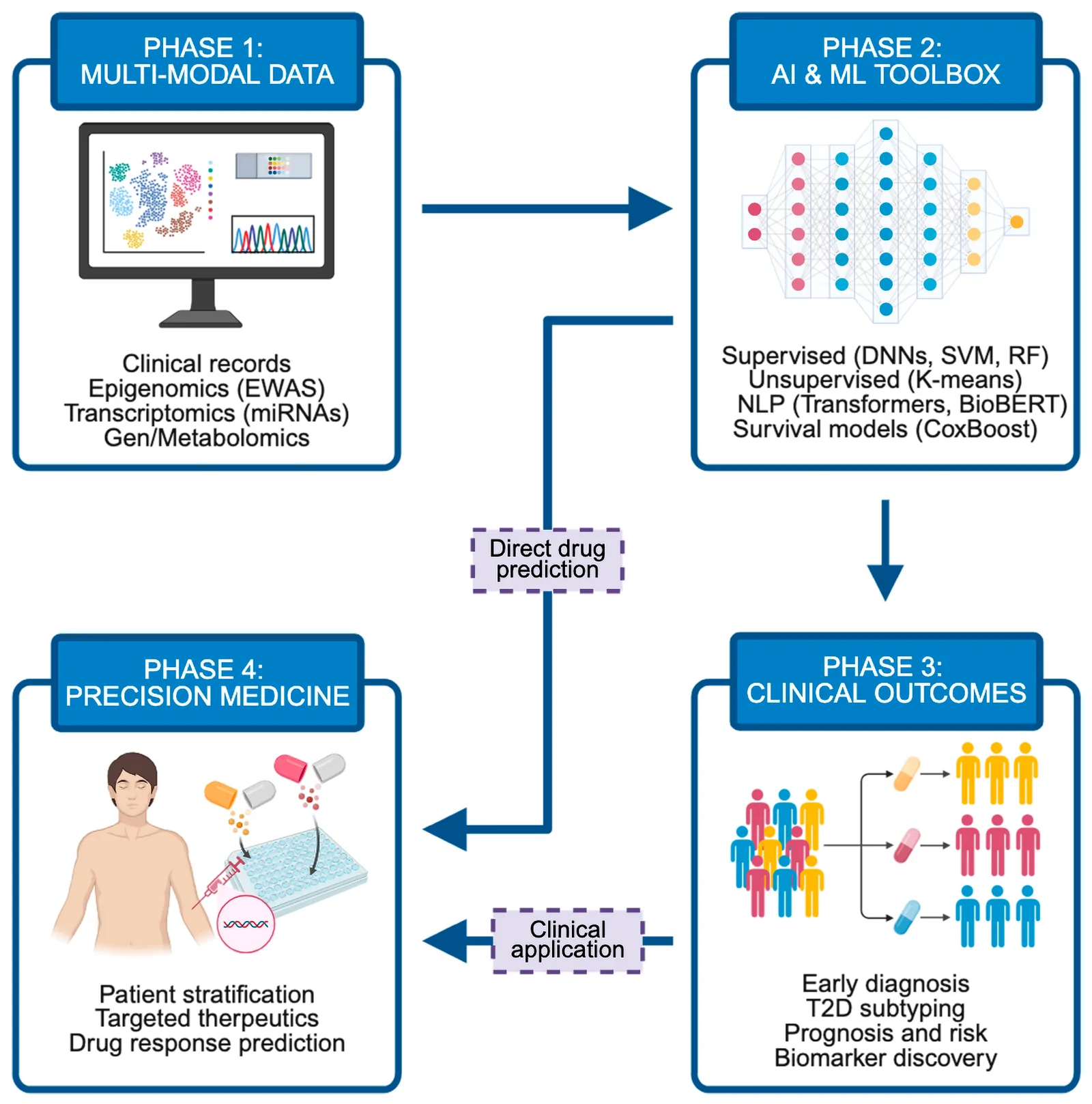
AI and ML-driven workflow for Type 2 Diabetes: from multimodal data integration to precision medicine. The flowchart outlines a four-phase pipeline for implementing artificial intelligence (AI) and machine learning (ML) in T2D management. Phase 1 involves collecting multimodal data, including clinical records (EHRs), epigenomic, transcriptomic, and metabolomic profiles. In Phase 2, the AI & ML toolbox applies supervised and unsupervised learning, Natural Language Processing (NLP), and survival models to process these complex datasets. This computational engine powers two converging pathways: generating actionable Phase 3 clinical outcomes (such as early diagnosis, robust subtyping, and novel biomarker discovery) and predicting drug response directly. Both pathways culminate in Phase 4, enabling precision medicine through advanced patient stratification and targeted therapeutics.

**Table 1:** Main genes involved in the development and progression of type 2 diabetes.

Gene	Mechanisms of Action Involved in T2D Progression	Chromosome	SNP	SNP Functional Effect	Reference
***HNF1B***	Encodes transcription factor HNF-1β, which regulates β-cell development and insulin secretion	17q12	rs4430796	Modulates the expression of ***HNF1B*** through the binding of the transcription factor PAX6.	[59]
***WFS1***	Encodes wolframin; regulates ER Ca^2+^ homeostasis and ER stress; loss-of-function causes ER stress, β-cell dedifferentiation, and impaired insulin secretion	4p16	rs1046322	miR-668 binding site (reducing the amount of wolframin)	[60,61,62]
			rs9457	miR-668 binding site	
			rs10010131	Reduced insulin secretion (G allele)	
			rs1046320	No clear biological function	
***GCK***	Encodes glucokinase; acts as glucose sensor in pancreatic β-cells and regulates insulin secretion	7p15.3-p15.1	rs1799884	Modulates Glucokinase function, affecting Insulin secretion and glucose homeostasis	[63]
***GCKR***	Encodes glucokinase regulatory protein; inhibits hepatic glucokinase; reduces ***GCK*** inhibition: lower fasting glucose but increased triglycerides	2p23	rs780094	Reduces GKRP’s inhibitory binding to hepatic GCK, leading to lower fasting glucose but increased triglycerides, hepatic lipogenesis, and a paradoxical, population-dependent increase in T2D risk	[63,64]
***G6PC2***	Encodes islet-specific G6Pase (IGRP); opposes ***GCK*** by hydrolyzing G6P, raising the glucose threshold for insulin secretion	2q31.1	rs560887	Enhances splicing: higher ***G6PC2*** expression, increased ***FBG***; rs560887-A lowers ***FBG***; the G allele is associated with increased T2D risk in some populations	[63,65]
***CDKN2A*** ***CDKN2B***	Cell-cycle inhibitors that regulate β-cell proliferation	9p21	rs2383208	Leads to premature islet aging, reduced β-cell proliferation, and impaired insulin secretion; T2D risk association is population-dependent	[66,67]
***CDKAL1***	Encodes a methylthiotransferase that modifies tRNA for proinsulin translation	6p22.3	rs2206734	Affects insulin secretion and insulin resistance	[66]
***SLC30A8***	Encodes ZnT-8; transports zinc into insulin vesicles, stabilizing insulin and regulating secretion	8q24.11	rs13266634	A missense variant that boosts an overlapping enhancer, is associated with T2D and insulin resistance, and interacts with serum zinc levels.	[68]
***IGF2BP2***	Encodes an RNA-binding protein (m^6^A reader) that regulates translation and stability of mRNAs, including IGF2 and PDX1; modulates pancreatic β-cell proliferation and insulin secretion	3q27.2	rs4402960	Strongly associated with T2D	[69,70]
			rs6777038	Strongly associated with T2D	
			rs6444082	Shows nominal association with body fat and HOMA-IR.	
***PROX1***	Encodes a homeobox transcription factor critical for pancreatic development and β-cell function	1q32.2-q32.3	rs340874	Reduces ***PROX1*** expression, impairing glucose-stimulated insulin secretion, and is associated with higher fasting glucose and increased visceral fat accumulation	[71,72]
***ADAMTS9***	Encodes a secreted metalloprotease that degrades ECM proteoglycans	3p14.3-p14.2	rs4607103-	Increases ***ADAMTS9*** expression, impairing insulin sensitivity in skeletal muscle via integrin β1 signaling and mitochondrial dysfunction, thereby increasing T2D risk through insulin resistance rather than β-cell dysfunction	[73]
***IRS1***	Encodes IRS-1, a key adaptor protein in insulin signaling	2q36	rs10498210	Not yet fully clarified; it has been associated with an effect on IRS-1 protein function or its expression levels	[74]
***GCKR***	Regulates glucokinase activity in the liver and pancreatic islet cells; regulates glucose uptake and lipogenesis	2p23	rs780094	Reduces GKRP–GCK binding, increasing hepatic GCK activity, lower fasting glucose but increased triglycerides and NAFLD; T2D association is paradoxical, mediated by insulin resistance and long-term β-cell dysfunction.	[75]
***FTO***	Regulates body weight and metabolism. Involved in the oxidation of fatty acids and the regulation of energy balance	16q12.2	rs9939609 rs8050136	Obesity risk alleles that promote lipid storage via ***IRX3*/*IRX5*** upregulation; T2D risk is primarily mediated by increased BMI/insulin resistance	[76,77]

**Table 2:** Examples of the most used tools in the field of genomics and transcriptomics that integrate AI and ML.

Tool	Description	Application in Genomics	Platform
DeepVariant	Uses deep neural networks for variant calling in high-throughput sequencing data.	Precise identification of genetic variants (SNPs and indels).	Open-source, Linux, Docker
XGBoost	Efficient boosting algorithm based on decision trees.	Classification of genetic variants and prediction of complex phenotypes.	Python, R
scikit-learn	Python library for machine learning with classification and regression algorithms.	Variant classification and pattern searching in genomic data.	Python
TensorFlow and Keras	Libraries for building high-performance deep neural networks.	Advanced analysis of genetic sequencing data and disease prediction.	Python
Variant Effect Predictor (VEP)	Tool to score and predict the functional impact of genetic variants.	Functional annotation of variants before predictive modeling.	Web-based, API
Bio-Rad AI Solutions	Platform for analyzing genetic data with AI in the detection of rare mutations.	Accurate quantification of rare mutations in molecular diagnostics.	Specialized hardware
Hail	Framework for large-scale genomic data analysis, oriented towards big data.	GWAS analysis, genetic variants, and large-scale classification.	Python, Spark
Kallisto + Sleuth	Rapid quantification of gene expression and statistical analysis of RNA-Seq data.	Identification of differentially expressed genes and biomarkers.	Python (Kallisto), R (Sleuth)
CYP450 + ML Models	Drug interaction databases along with ML models for pharmacogenomics.	Predicting drug responses based on genetic variants.	Databases + ML Models
GenePattern	Bioinformatics platform for genetic analysis with AI/ML integration.	Classification of genomic data and pattern search in sequencing.	Web-based
GATK (Genome Analysis Toolkit)	Framework developed for processing sequencing data and detecting genetic variants with high precision.	Analysis of SNPs and indel variants in next-generation sequencing (NGS) data.	Java, Open-source
PLINK	Toolkit for the analysis of genotypes and phenotypes in genetic association studies.	GWAS analysis, heritability estimation and variant filtering.	C/C++, Linux, MacOS, Windows
AutoML (Google Cloud AutoML)	Platform that enables the automated development of custom machine learning models without the need for programming experience.	Development of predictive models for genomic analysis and variant classification.	Web-based, API, Google Cloud
BioBERT	Pre-trained natural language model for processing biomedical literature.	Analysis of scientific literature to extract relationships between genes, diseases and variants.	TensorFlow, PyTorch, Python
Monocle	Software for the analysis of single-cell transcriptomic data (scRNA-seq).	Analysis of cellular trajectories and genetic dynamics in single-cell studies.	R, Bioconductor

## Data Availability

No new data were created or analyzed in this study.
